# Maternal Stress Responses and Coping Following a NICU Experience

**DOI:** 10.3390/children12060660

**Published:** 2025-05-22

**Authors:** Kim K. Doheny, Fumiyuki C. Gardner, Saher Ali, Brittany J. Fronheiser, Claire J. Miller, Gina M. Brelsford

**Affiliations:** 1 Department of Neural and Behavioral Sciences, Penn State College of Medicine, Hershey, PA 17033, USA; kkh1@psu.edu; 2 Department of Pediatrics, Penn State College of Medicine, Hershey, PA 17033, USA; fumiyuki.c.gardner@gmail.com (F.C.G.); saher.ali122@gmail.com (S.A.); brittany.fronheiser@pennmedicine.upenn.edu (B.J.F.); cmiller49@pennstatehealth.psu.edu (C.J.M.); 3 School of Behavioral Sciences and Education, Penn State Harrisburg, Middletown, PA 17057, USA

**Keywords:** stress, NICU, maternal, coping, salivary cortisol, heart rate variability

## Abstract

**Background/Objectives**: Mothers of infants admitted to the neonatal intensive care unit (NICU) experience significant stress, which can have lasting effects on mental health and parent–infant bonding. This mixed-methods study aimed to explore maternal stress response, coping, and resilience by examining physiological stress markers and maternal narratives. **Methods**: A total of 28 mothers who had an infant hospitalized in the NICU within the past three years participated in a two-hour laboratory session, which included stress induction using the Trier Social Stress Test (TSST). Salivary cortisol (sCort) and heart rate variability (HRV) were measured to assess physiological responses. **Results**: Qualitative analysis of maternal narratives identified two distinct response patterns: an anger/trauma (AT) group (*n* = 7) and a gratitude/optimism (GO) group (*n* = 6), with the remaining 15 mothers classified as a mixed (M) group. GO mothers exhibited significantly higher cortisol reactivity during recovery compared to AT mothers (*p* < 0.01). While GO mothers had higher baseline HF-HRV, no significant between-group differences were found in HRV responses. **Conclusions**: Findings suggest that maternal perception of NICU experiences is associated with distinct physiological stress response patterns, highlighting the importance of stress appraisal and coping in maternal well-being.

## 1. Introduction

In 2023, approximately 9.8% of infants born in the United States of America were admitted into the neonatal intensive care unit (NICU) for reasons such as prematurity, transient tachypnea of the newborn, respiratory distress syndrome, jaundice, sepsis, and necrotizing enterocolitis [1]. In addition to the concern for their medically vulnerable infants, parents of infants in the NICU face many uncomfortable stressors, including unfamiliar surroundings, highly technical atmosphere, and dependence on healthcare providers [2]. Some mothers may struggle with role identity in caring for their infant when the maternal–infant bond is disrupted by NICU admission [3,4,5]. Moreover, many parents do not perceive themselves as capable of taking home their medically complex infant at discharge [6]. Even long after the NICU discharge, having an infant that needs extensive medical treatment affects new parents [7,8]. Parents of medically complex infants report greater financial burden, impact on family and social functioning, and increased perceived stress [8,9,10,11].

Stress and mental health management are crucial for families because unmanaged stress, anxiety, and depression can lead to disrupted bonding with their infants [12]. Therefore, it is important to identify mothers’ perceptions and stress reactivity to determine how well they are coping with stress. Lazarus and Folkman’s Transactional Stress and Coping theory states that the way a person interprets an event will influence how they react [13]. According to this theory, first, a stressor is cognitively appraised and then that appraisal influences one’s coping, which mediates an emotional response [13]. Stress was first defined as an engineering term to explain the forces that objects experienced, and we have adapted the term *stress* in humans to explain the many forces we feel [14]. Coping is defined as using cognitive and behavioral efforts to manage specific stressors [13].

Similarly, allostasis is defined as the process the body uses to respond to physiological and psychological stressors to return to homeostasis [15]. The body relies on the hypothalamic–pituitary–adrenal (HPA) axis to regulate responses to stress through the mediation of hormones.

One of the most studied biomarkers of psychosocial stress response is salivary cortisol (sCort). When stress is perceived, corticotropin-releasing hormone (CRH) is released by the paraventricular nucleus (PVN) of the hypothalamus. In a cascading manner, the anterior pituitary releases adrenocorticotropic releasing hormone (ACTH), which then triggers the adrenal glands to secrete cortisol, a stress hormone [14,16]. Salivary cortisol levels increase within a few minutes following stimulation and reach peak concentrations 10–30 min after cessation [17]. Cortisol can cross the blood–brain barrier and access receptors in the hippocampus, amygdala, and frontal lobes, which are implicated in processing memory and learning [14,18].

Another widely used method to measure physiological response to stress is heart rate variability (HRV). HRV is the measure of the electrophysical variance between each heartbeat, and it can be analyzed by time and frequency domains. The key system that influences heart rate is the central autonomic network, comprised of the cortical, limbic, and brain stem regions, which innervates the autonomic nervous system (ANS) [19]. The ANS has two branches, the sympathetic nervous system (SNS), responsible for increasing heart rate, breathing, pupil constriction, and slowing digestion, and the parasympathetic nervous system (PNS), responsible for the antagonistic effect of the SNS [19]. The latencies between the actions of these systems produce oscillations in heart rate and using frequency analysis, HRV can be divided into two components: low-frequency band (LF-HRV), which reflects both SNS and PNS innervation, and high-frequency band (HF-HRV), which primarily reflects respiration and cardiac parasympathetic tone [19,20,21].

HF-HRV can be used as an indicator of a person’s resilience to stress. Resilience can mean achieving a positive outcome in the face of adversity or active resistance to a stressor [22]. High levels of resting HRV suggest high emotional regulation. According to a study by Denson, Grisham, and Moulds on the effects of various self-regulation strategies on HRV, the group that used cognitive reappraisal in response to an angering stimulus reported higher HRV compared to the control and emotion suppression groups [23]. This study also suggested that suppression of emotion in response to an angering stimulus was correlated to higher HRV than the control, but lower HRV compared to the reappraisal group [23]. Another study performed by An et al. with soldiers in the Army National Guard Special Forces found that greater stress resilience was associated with parasympathetic dominance during resting periods [20].

Given the importance of understanding maternal stress response, coping, and resiliency, we used a combination of qualitative and quantitative methods to depict maternal perspectives and physiological responses to their NICU experience. Using a mixed-methods approach, qualitative robust individual narratives [24] can be paired with quantitative responses, and both methods can be used to validate findings [25]. The aim of this study was to describe maternal patterns of response to psychological stress and explore the associations among perception and recall of mothers’ NICU experience with their biophysiological measures of stress response and resiliency. We hypothesized that mothers would show increased stress response to recall and narration of their NICU experience and expected to find an inverse association between vagal tone (resilience) and cortisol reactivity.

## 2. Materials and Methods

Participants (N = 28) were comprised of a convenience sample of mothers who were recruited using flyers, social media, and phone solicitation within a 50-mile radius of the research laboratory located in southcentral Pennsylvania. All participants were healthy mothers over the age of 18 who spoke fluent English and had an infant hospitalized in the NICU in the past three years. Mothers were prescreened and excluded from the study if there was presence of an immune, cardiovascular, endocrine, or previously diagnosed psychiatric condition known to influence psychophysiological data acquisition. The current protocol was conducted in accordance with the Declaration of Helsinki standards and approved by the medical center’s affiliated institutional review board.

### 2.1. Procedure

Participants were scheduled for a two-hour psychophysiological observation in the research laboratory between the hours of noon to 6:00 pm. Observation included periods of acclimation, stress induction, and recovery.

Following written informed consent, participants sat quietly for five minutes prior to collection of the initial saliva sample. After rest, three surface electrocardiogram electrodes were placed on the participants’ chest and lower abdomen using standard bipolar lead II placement for continuous heart rate variability (HRV) measurement during the ten minutes of baseline, fifteen minutes of stress induction, and twenty minutes of recovery. Throughout the observation period, participants were instructed to sit upright with their feet on the floor, legs uncrossed, and hands placed on their thighs in order to limit motion artifacts.

Following baseline, the Trier Social Stress Test (TSST), a gold-standard approach to stress induction in laboratory settings [26,27,28], was administered. The TSST consists of periods of preparation, task performance, and recovery [28,29]. Mothers were given five minutes to mentally prepare a narrative describing their experiences as a mother of a newborn infant who required NICU hospitalization. They were then prompted to recount their prepared personal stories for five minutes. Probes were used to clarify instructions (e.g., “Feel free to talk about any aspect of your NICU experience that you would like to describe”) or to prompt participants to continue talking for the five-minute duration. The narratives were audio-recorded and later transcribed verbatim. Finally, the mothers performed a verbal arithmetic task for five minutes (i.e., starting at 1022, sequentially subtract the number 13 and report your answers out loud) where they were instructed to restart from the beginning if they gave an incorrect response.

A 40 min period of stress recovery followed the TSST, during which mothers sat quietly, relaxed, and avoided engagement with written or digital media. Questionnaires asking about their distress in the past week were administered at 20 min post-stress induction after removal of electrodes. Free-flow saliva samples were collected at 20 and 40 min post-stress induction. Prior to leaving the lab, participants were debriefed and provided stress/coping support literature and awarded monetary compensation for their time and travel to the lab of 50 US dollars. Assessment of additional support or referral was made by phone contact one week following observation.

### 2.2. Measures

#### Salivary Stress Hormones

In order to prevent interference with the immunoassay, participants were instructed to avoid eating, drinking, and smoking one hour prior to their laboratory appointment. Saliva samples (1 mL aliquots) were collected in SalivaBio Passive Drool tubes and stored at −80 °C until being sent to Salimetrics^®^ SalivaLab (Carlsbad, CA, USA) for analysis of salivary cortisol (sCort). All samples were assayed in duplicate on the same plate using Salimetrics^®^ Salivary Cortisol Assay Kit (Cat. No. 1-3002) as per the manufacturers’ protocol. Inter- and intra-assay coefficients of variation were below 6%, which met the manufacturer’s criteria for accuracy and repeatability in Salivary Bioscience and exceeded the applicable NIH guidelines for Enhancing Reproducibility through Rigor and Transparency [30].

### 2.3. Heart Rate Variability

Heart rate variability (HRV) was measured using MindWare^®^ Mobile Impedance Cardiograph (MindWare^®^ Technologies, Gahanna, OH, USA) and was later analyzed using the MindWare^®^ HRV analysis software (version 3.1.6). For this study, cardiac vagal tone, as measured by high-frequency HRV (HF-HRV), was the variable of interest for analysis. Using Fast Fourier Transform (FFT), spectral analysis was conducted for HF-HRV (0.15–0.40 Hz) bandwidth using 60 s segments of R-R wave data collected between baseline and recovery phases. Each R-R wave segment was manually screened to be free from movement artifact and ectopic beats prior to analysis.

### 2.4. Current Health Status

Maternal health status was assessed using Touchette’s Health Survey and Medication List questionnaire [31]. This screening questionnaire contains 11 items regarding date of birth, biological sex, current health diagnoses, medications, health service usage, and menstrual cycle [31]. All mothers were required to complete the questionnaire.

### 2.5. Perceived Stresss

Maternal stress was assessed using the Perceived Stress Scale (PSS) and Distress Thermometer—Parent (DT-P) questionnaires. The PSS is a 10-item, widely used measure of non-specified perceived stress an individual experienced within the past month [32]. Example questions include, “In the last month, how often have you felt nervous and ‘stressed’?” and “In the last month, how often have you felt that you were unable to control the important things in life?” Scores range from 0 to 40, with a higher score indicating greater stress perception in one’s life [32].

The DT-P includes a visual analog scale depicting a thermometer, for which the participant circles their level of distress in the past week between 0 (no distress) and 10 (extreme distress). Levels above 4 are recommended for mental health service referral [33]. The DT-P also contains a dichotomous (yes/no) problem list in six domains: practical (7 items), social (4 items), emotional (9 items), physical (7 items), cognitive (2 items), and parenting problems (7 items if child < 2 years of age, 5 items if child > 2 years of age) commonly experienced within the past week [33]. The number of “yes” responses were added for each subcategory (e.g., 7 out of 9 emotional score) and for an overall score (range 0 to 34/36 based on the age of infant). All mothers completed the two perceived stress questionnaires without any missing responses.

### 2.6. Statistical Analysis

Qualitive data (mothers’ narratives of NICU experiences) were analyzed using narrative inquiry followed by thematic content analysis. During narrative inquiry, authors independently analyzed and interpreted the data one narrative at a time by contemplating the words of the transcripts and taking note of the characters, emotions, memories, complexity, and cohesiveness of the entire interview [7,34]. Each mother’s NICU experience was then briefly summarized. Triangulation amongst study team members, led by the principal investigator, consisted of numerous collaborative discussions to comprehensively confirm and share the different perspectives necessary to gain a greater understanding of each narrative, validate each of their meanings, and review existing literature [35].

During the thematic content analysis, the brief summaries that emerged from each narrative facilitated the development of common themes and concepts that collectively described mothers’ NICU experiences [36]. These themes, along with each narrative’s personal characteristics, informed the categorization of maternal narratives into anger/trauma (AT), gratitude/optimism (GO), or mixed (M) groups. The AT group included narratives with defining characteristics such as guilt, anger, perseverance, and vivid recounting of experiences. Narratives that displayed logical flow, gratitude, reframing of negative experiences, and progression from past trauma were placed into the GO group. Narratives that were not consumed by characteristics pertaining to trauma, anger, gratitude, or optimism made up the M group.

SPSS version 27 (IBM Corporation, Armonk, NY, USA) was used for quantitative analyses. Descriptive statistics were computed for salivary cortisol (sCort) and HF-HRV to check for skewness and outliers, and sCort and HF-HRV were naturally log transformed prior to analyses. For each group (i.e., AT, GO, M), paired sample *t*-tests were used to determine between-phase (i.e., baseline, 20, and 40 min recovery) differences in sCort, and between-phase (i.e., baseline, stress induction, 20 min recovery) differences in HF-HRV. Change scores were calculated between two phases (e.g., baseline and stress induction, 20 and 40-min recovery), and their group differences were examined with independent samples *t*-test. Group differences in perceived stress were also examined using independent sample *t*-tests. All tests were two-tailed at a 5% significance level.

## 3. Results

Participants were majority (89%) white/non-Hispanic mothers with a mean (SD) age of 30.7 (5.4) years, whose former NICU infants were 16.5 (8.9) months at the time of study session. On the Health Survey and Medication List Questionnaire, seven mothers (25%) reported having depression, two mothers (7%) reported having asthma and/or high blood pressure, and 4% of mothers reported presence of one of the diagnoses including diabetes, memory problem, and chronic pain. Sixty-eight percent of the mothers were not taking any medication at the time of study session, 14% of the mothers were on birth control, 14% of the mothers had a change in medication in the past month, and 7% of the mothers started seeing a new doctor, were seen in an emergency room, and/or were discharged from a hospital within the past month.

### 3.1. Thematic Analysis of Narratives

Using qualitative analysis of the mothers’ narratives, three categories emerged. In one subset of mothers, there were overarching themes of anger and guilt and the narratives were characterized by vivid descriptions, with traumatic recall and perseveration around events. We labeled this group the anger/trauma (AT) group (*n* = 7).

Some exemplar quotes from narratives to illustrate for the anger/trauma were as follows:

“Feeling like I was always in the way…having to move the rocking chair to my daughter’s bassinette every day, then when I would leave and come back it would be moved to another area”.

One mother expressed feelings of powerlessness:

“Just doing everything that I could to make sure everything was right and it just still doesn’t come out right”.

Another mother expressed uncertainty and anger for miscommunication:

“No one told us for 4 h that he was even taken back to the NICU. No one told us where he was, no one could tell us how our son was doing”.

Anger and trauma also included feelings of resentment for the inability to care for the infant due to being away from them:

“…they got to do everything that I didn’t get to do like you know feed him and everything else when I wasn’t there”.

Mothers expressed anger and trauma in various ways, such as powerlessness, uncertainty, and resentment. These mothers’ recall of NICU experiences appeared to focus on the challenges they experienced that illustrate their narrative.

In a second subset of mothers, there were overarching themes of gratitude and positive connections with nurses. These narratives were characterized by a logical progression through events, moving through trauma, reframing from negative to positive while making sense of a challenging situation. We labeled this group the gratitude/optimism (GO) group (*n* = 6).

In the gratitude/optimism group, some exemplar quotes were as follows:

“One thing we did look forward to is they almost had like a feeder grower room and the goal was to get there. Once you were there the goal was the next step was to get home. So there was almost hope inspired in that”.

“She really was in good hands … just extremely grateful for the nursing staff that was there and they really just became like a second family”.

“It was just nice to also have that community of other parents and families around you that were going through maybe even harder situations than I was going through. I was always very thankful and very appreciative … that when I did have to leave even though it was hard I knew that she was in the best care and best hands”.

The remainder of mothers did not distinctly fall into either category and were labeled the mixed group (*n* = 15). The current study focused on AT and GO groups to explore the relationship between these two distinct styles of recalling traumatic experiences with maternal stress and resiliency.

### 3.2. Sample Characteristics Among the Narrative Groups

Group comparison showed that mothers in the GO group were significantly older [34.2 vs. 27.1 years, *t* (11) = −2.63, *p* = 0.02], and their infants were also significantly older at the time of study session [21.4 vs. 10.7 months, *t* (10) = −2.45, *p* = 0.03], relative to the mothers in the AT group.

No significant group differences in medical problems were reported on the Health Survey and Medication List Questionnaire. However, three out of seven mothers (43%) in the AT group reported having depression, and one out of six mothers (17%) in the GO group reported having depression.

### 3.3. Physiological Stress Response

#### sCort

**Figure 1:**
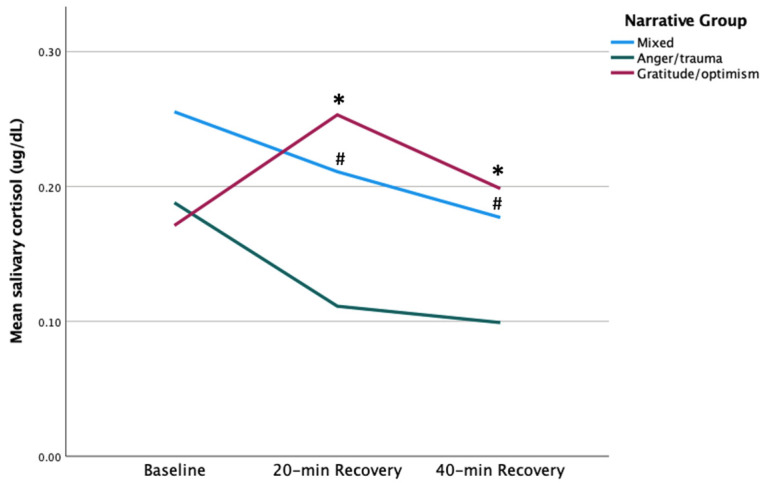
sCort maternal group comparison. # *p* < 0.05, * *p* = 0.01.

Mothers in the gratitude/optimism (GO) group showed peak salivary cortisol (sCort) at 20 min recovery, whereas mothers in anger/trauma (AT) and mixed (M) groups showed declining sCort responses from baseline to 40 min recovery (Figure 1). GO mothers’ sCort at 20 min recovery was more than twofold higher compared to AT mothers’ [0.25 vs. 0.11, respectively. *t* (11) = −3.05, *p* = 0.006]. Similarly, GO mothers’ sCort at 40 min recovery was higher compared to AT mothers’ [0.20 vs. 0.10, respectively. *t* (11) = −3.34, *p* = 0.003]. A repeated measures ANOVA with Greenhouse–Geisser correction with only AT and GO mothers showed no main effect of time or group; however, there was a significant time X group interaction effect that showed AT and GO mothers differed in patterns of sCort response from baseline to 40 min recovery, *F* (1.373, 15.107) = 8.80, *p* = 0.006. Mothers in the GO group showed higher sCort at 20-min, *t* (11) = 3.05, *p* = 0.01, and at 40 min recovery, *t* (11) = 3.34, *p* = 0.01, compared to mothers in the AT group. Similarly, mothers in the M group showed higher sCort at 20-min, *t* (20) = 2.28, *p* = *0*.03, and at 40 min recovery, *t* (20) = 2.27, *p* = 0.04, compared to mothers in the AT group. There were no significant differences between the mothers in M and GO groups at any time point.

**Figure 2:**
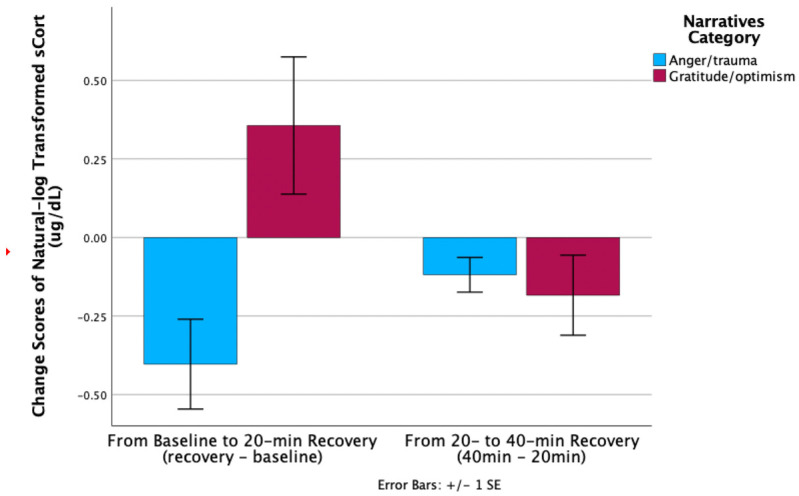
sCort recovery maternal group comparison.

Change scores from baseline to 20 min recovery were higher in AT vs. GO [*t* (11) = −2.99, *p* = 0.01] (Figure 2). Specifically, AT’s sCort declined from baseline to 20 min recovery (0.19 to 0.11), whereas GO’s sCort increased from baseline to 20 min (0.17 to 0.25). No significant group difference was found in sCort change score from 20 to 40 min recovery.

### 3.4. HF-HRV

**Figure 3:**
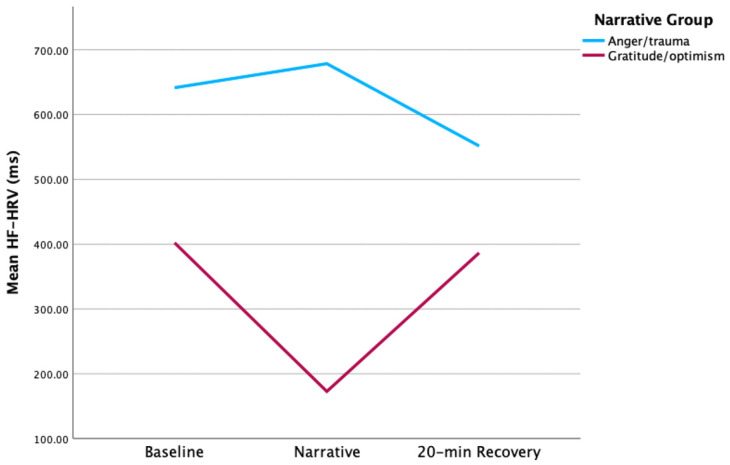
HF-HRV maternal group comparison.

**Figure 4:**
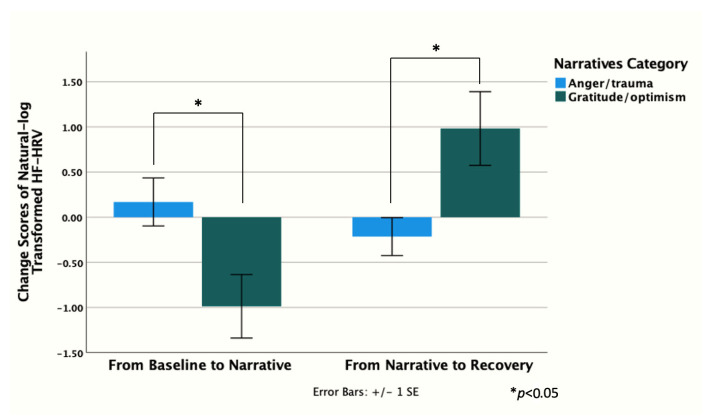
HF-HRV recovery maternal group comparison.

GO mothers showed higher HF-HRV at baseline and post-stress vs. AT; however, this difference was not significant (Figure 3). Similarly, no significant between-group differences in HF-HRV were found at any other point. GO mothers’ HF-HRV declined with stress, while AT mothers’ HF-HRV peaked during stress. Change scores from baseline to narrative, and narrative to post-stress were higher in GO vs. AT [*t* (7) = −2.56, *p* = 0.04 and *t* (8) = 2.79, *p* = 0.02, respectively] (Figure 4). Moreover, HF-HRV increased from narrative to post-stress [*t* (11) = 2.29, *p* = 0.04] for GO only. AT reported higher distress in the practical subdomain (i.e., housing, work, finances, housekeeping, transport, childcare, relaxing) compared to GO [2.57 vs. 0.83, *t* (11) = 2.29, *p* = 0.04].

### 3.5. Associations Between sCort and HF-HRV

Examinations of the associations between sCort and HF-HRV showed that in all mothers, higher baseline sCort was significantly associated with lower baseline HF-HRV [*r* (26) = −0.49, *p* = 0.01]. Similarly, higher baseline sCort was associated with lower HF-HRV at 20 min recovery [*r* (27) = −0.43, *p* = 0.03]. Additionally, lower HF-HRV during the narrative phase was associated with higher sCort at 40 min recovery [*r* (25) = −0.48, *p* = 0.01]. When the associations between sCort and HF-HRV were examined separately by AT and GO, no significant associations were found in GO. However, in AT, significant associations emerged concurrently at baseline and 20 min recovery, and subsequently, from HF-HRV measured earlier to sCort measured at 20 and 40 min recovery or vice versa. Importantly, all associations were inverse associations between sCort and HF-HRV (i.e., higher sCort, lower HF-HRV).

## 4. Discussion

This study aimed to examine the associations among maternal perceptions of their infants’ NICU hospitalization, and their salivary cortisol (sCort) and high-frequency heart rate variability (HF-HRV) responses to the stress induction, which included recounting of their infants’ NICU hospitalization and discharge. Although some mothers’ experiences were over two years prior to the study session, qualitative examinations of their narratives revealed strong impressions of their infants’ NICU hospitalization on their memories. These impressions evidenced the traumatic nature of the NICU experience for some mothers.

Numerous longitudinal studies have looked at parents’ perceptions of their NICU experience [2,6,37]. In a study performed by Haemmerli et al., they found that parents struggled with the loss and recovery of expectation in having a healthy child. These struggles were reflected in the parents’ reports of feeling destabilized, helpless, overwhelmed, and alienated [2]. Even after the infant’s NICU discharge, the stress and emotions do not disappear. Multiple studies showed that increased infant health issues while in the NICU contributed to increased familial burden after discharge [38,39]. Another study found that at 14 months post-partum, mothers showed evidence of depressive and traumatic symptoms [40]. In a smaller study, parents indicated the word “trauma”, as opposed to “stress” more adequately described experience of preterm birth [41].

While these studies showed parents’ trauma from their NICU experiences, our qualitative examination of the mothers’ narratives sheds light on the differences in the degree to which they have overcome this trauma. Specifically, some mothers vividly described their NICU experiences with anger and guilt with traumatic recall around events (i.e., AT mothers). On the other hand, some mothers described their experiences with gratitude and positive connections with nurses. Their narratives showed logical progression through events, and their processes of making sense of the challenging situation through reframing (i.e., GO mothers). Notably, we found that these two distinct styles of recall were reflected on the mothers’ biophysiological responses to stress induction.

The mothers’ sympathetic stress responses were measured using salivary cortisol. As hypothesized, GO mothers’ sCort increased in response to stress and decreased as they recovered from the stress induction. On the other hand, AT mothers showed dysregulation in the pattern of change in sCort, with continuing decrease from baseline through 40 min post-stress induction. Notably, AT mothers’ sCort decrease from baseline to 20 min post-stress induction was higher in magnitude compared to GO mothers’ sCort increase in the same period. Although sCort has frequently been used to examine stress in infants, it has rarely been used to assess stress in mothers of infants requiring admission.

AT mothers’ dysregulation in biophysiological pattern of response to stress induction was also observed in HF-HRV. Although no difference between AT and GO mothers in HF-HRV values were observed at any point, there were differences in the pattern of change from baseline to recovery. In GO mothers, the lowest point in HF-HRV occurred during narrative. On the other hand, AT mothers’ HF-HRV stayed level throughout the session. The HF-HRV pattern seen in GO mothers aligns with the concept of vagal break developed by Porges [42]. This concept posits that myelinated vagus functions to rapidly inhibit or disinhibit vagal tone of the heart, thereby mobilizing or calming an individual based on the context. The decrease in HF-HRV among GO mothers during stress (i.e., recounting the NICU experience) may suggest inhibition of vagal tone to mobilize sympathetic response. The subsequent increase in GO mothers’ HF-HRV during recovery may suggest disinhibition of vagal tone to encourage calmness and recovery after the source of stress has passed. This pattern of HF-HRV that suggests vagal break was not seen in AT mothers, which, in alignment with their sCort activity, may point to their dysregulation in stress response.

Studies have shown that there can be stability in HRV while sCort shows fluctuation over the same time period. A study performed by Mazgelyte et al. showed stability of HRV over three data points across 5 days, but variability in sCort over the same time period [43]. Distress and anxiety disorders are marked by blunted and inflexible patterns of autonomic reactivity and do not allow for adequate adaptability to stressors [44]. In addition to the pattern of change in HF-HRV and sCort, the current study also examined the associations between these two stress response measures. We found negative associations between sCort and HF-HRV; however, probing of these associations revealed they were only seen in AT mothers and not GO mothers. The negative association between baseline HF-HRV and sCort is similar to Mazgelyte et al., who found a significant negative relationship between time-domain HRV indices (RMSSD, pNN50) and sCort in baseline conditions in healthy non-stressed and moderately stressed samples [43]. Higher resting cardiac vagal tone in a healthy sample of men and women was linked with TSST-induced increases in cortisol [45]. There was also a study that reported lower cortisol response to a stressor and a faster change in heart rate, indicating an inverse association between vagal tone and HPA axis in response to stress [46].

Our findings, albeit capturing a small sample of mothers who represent a specific cross-section of NICU mothers, provides opportunities for further understanding of the intersections among maternal perception, stress, and resilience. Particularly, the dysregulations in sCort and vagal withdraw seen in AT mothers can be probed using longitudinal studies with prenatal and postnatal measurements of sCort and HF-HRV. Changes in these measurements may give additional insight into whether certain mothers, such as those with dysregulated sCort responses or lower HF-HRV, are more vulnerable to perceiving and experiencing trauma, or if traumatic experiences, such as their infant’s NICU admission, negatively impacts their stress response and resiliency. Another factor to consider is consideration of menstrual cycle, which has been shown to affect sCort response. Specifically, menstrual phase affected cortisol levels but not perceived stress [47]. Cortisol levels were higher in the follicular phase in response to the TSST when the hormones progesterone and estradiol were lower, compared to the luteal phase where the cortisol response was lower, and the hormones were higher [47].

### 4.1. Limitations

This exploratory study was one of the first to investigate associations between mothers’ perceptions of their NICU experiences and physiological stress response patterns. However, as with all research, there are notable limitations to this study. Firstly, we were able to secure a small sample of participants via convenience sampling. If our sample size was larger, although not common in qualitative research, there may be more generalizability of findings. Additionally, the manner of acquiring participants for this study via flyers and personal contacts with mothers who were in the local NICU, can also affect the generalizability of findings. Although patients at a research hospital are often approached to engage in research, there may be distinct differences between those who agree to study participation and those who do not. For instance, those who offer to participate may have additional means of coping and supports that are more effective, thereby allowing them the mental bandwidth to engage in a study following their NICU experience. Moreover, engaging in an in-person lab-based setting is not possible or desirable for all former NICU mothers and, therefore, this sample may possess unique qualities related to resilience and coping that we were unable to assess. Further, use of the Trier Social Stress Test (TSST) elicits a unique type of stress that is not reflective of all stress-inducing situations. Thus, although a gold standard in use of stress induction in an artificial laboratory setting, the manner of stress the TSST invoked may be unique to this laboratory setting and may not generalize to other inducers of life stress for mothers in this sample. Lastly, the time since birth to engagement in this study may have an impact on attenuation of mothers’ stress responses.

### 4.2. Conclusions

Although there are limitations to this study, exploration of mothers’ qualitative narratives of stress and coping and biological markers of stress was a worthwhile aim of this exploratory study. As noted above, stress in NICU mothers has been examined in the literature through qualitative studies and physiological measures of stress. However, there has been no study showing associations between qualitative characteristics of mothers’ narratives and their physiologic stress response. Our study demonstrates how the cognitive processing and interpretation of the NICU experience/trauma may have long-term impacts on physiologic handling of stress. HRV may be the mechanism between the appraisal and integration of a stressor and guides the physiological response [48]. This provides a potential target for interventions aimed at improving maternal coping and health. Families may benefit from discharge follow-up discussions to better inform parents on infant health and care, increase their confidence and knowledge, decrease their stress, and increase their feeling of social support [49]. Some successful interventions aimed at reducing depressive and post-traumatic stress symptoms in mothers of preterm babies have focused on improving mothers’ knowledge of and engagement in caring for their infants [50]. Our findings suggest that helping mothers to articulate and reframe trauma to optimism and hopefulness may support higher vagal tone and stress resilience. Thus, medical care providers should embrace opportunities for building stress resilience in parents through facilitative discussion and stress-processing of the NICU experience during hospitalization and post-discharge.

## Data Availability

Data are unavailable due to privacy or ethical restrictions.

## Abbreviations

The following abbreviations are used in this manuscript:

HRV	Heart Rate Variability
HF-HRV	High-Frequency Band Heart Rate Variability
sCORT	Salivary Cortisol
GO	Gratitude/Optimism Maternal Responses
AT	Anger/Trauma Maternal Responses
M	Mixed Maternal Responses

## References

[1] Martin J.A., Osterman M.J.K. (2025). Increases in Neonatal Intensive Care Admissions in the United States, 2016–2023.

[2] Haemmerli N.S., Lemola S., Holditch-Davis D., Cignacco E. (2020). Comparative Evaluation of Parental Stress Experiences Up to 2 to 3 Years After Preterm and Term Birth. Adv. Neonat. Care.

[3] Neu M., Klawetter S., Greenfield J.C., Roybal K., Scott J.L., Hwang S.S. (2020). Mothers’ Experiences in the NICU Before Family-Centered Care and in NICUs Where it Is the Standard of Care. Adv. Neonat. Care.

[4] Baia I., Amorim M., Silva S., Kelly-Irving M., de Freitas C., Alves E. (2016). Parenting very preterm infants and stress in Neonatal Intensive Care Units. Early Hum. Dev..

[5] Fegran L., Helseth S., Fagermoen M.S. (2008). A comparison of mothers’ and fathers’ experiences of the attachment process in a neonatal intensive care unit. J. Clin. Nurs..

[6] Adama E.A., Bayes S., Sundin D. (2018). Parents’ experiences of caring for preterm infants after discharge with grandmothers as their main support. J. Clin. Nurs..

[7] Adkins C.S., Doheny K.K. (2017). Exploring Preterm Mothers’ Personal Narratives Influences and Meanings. Adv. Nurs. Sci..

[8] Treyvaud K., Lee K.J., Doyle L.W., Anderson P.J. (2014). Very Preterm Birth Influences Parental Mental Health and Family Outcomes Seven Years after Birth. J. Pediatr..

[9] Balakrishnan A., Stephens B.E., Burke R.T., Yatchmink Y., Alksninis B.L., Tucker R., Cavanaugh E., Collins A.M., Vohr B.R. (2011). Impact of very low birth weight infants on the family at 3 months corrected age. Early Hum. Dev..

[10] Cronin C.M.G., Shapiro C.R., Casiro O.G., Cheang M.S., Math M. (1995). The Impact of Very-Low-Birth-Weight Infants on the Family Is Long-Lasting—A Matched Control Study. Arch. Pediat. Adol. Med..

[11] Zerach G., Elsayag A., Shefer S., Gabis L. (2015). Long-Term Maternal Stress and Post-traumatic Stress Symptoms Related to Developmental Outcome of Extremely Premature Infants. Stress Health.

[12] Bernardo J., Rent S., Arias-Shah A., Hoge M.K., Shaw R.J. (2021). Parental Stress and Mental Health Symptoms in the NICU: Recognition and Interventions. Neoreviews.

[13] Folkman S., Lazarus R.S. (1988). The Relationship between Coping and Emotion—Implications for Theory and Research. Soc. Sci. Med..

[14] Lupien S.J., Maheu F., Tu M., Fiocco A., Schramek T.E. (2007). The effects of stress and stress hormones on human cognition: Implications for the field of brain and cognition. Brain Cogn..

[15] McEwen B.S. (2002). Introduction—Protective and damaging effects of stress mediators: The good and bad sides of the response to stress. Metabolism.

[16] Thau L., Gandhi J., Sharma S. (2023). Physiology, Cortisol. StatPearls.

[17] Foley P., Kirschbaum C. (2010). Human hypothalamus-pituitary-adrenal axis responses to acute psychosocial stress in laboratory settings. Neurosci. Biobehav. R.

[18] Lupien S.J., Wilkinson C.W., Brière S., Ng Ying Kin N.M.K., Meaney M.J., Nair N.P.V. (2002). Acute modulation of aged human memory by pharmacological manipulation of glucocorticoids. J. Clin. Endocr. Metab..

[19] Appelhans B.M., Luecken L.J. (2006). Heart rate variability as an index of regulated emotional responding. Rev. Gen. Psychol..

[20] An E., Nolty A.A., Amano S.S., Rizzo A.A., Buckwalter J.G., Rensberger J. (2020). Heart Rate Variability as an Index of Resilience. Mil. Med..

[21] Task Force of the European Society of Cardiology and the North American Society of Pacing and Electrophysiology (1996). Heart rate variability: Standards of measurement, physiological interpretation and clinical use. Circulation.

[22] McEwen B.S., Gray J.D., Nasca C. (2015). Recognizing resilience: Learning from the effects of stress on the brain. Neurobiol. Stress.

[23] Denson T.F., Grisham J.R., Moulds M.L. (2011). Cognitive reappraisal increases heart rate variability in response to an anger provocation. Motiv. Emot..

[24] Renjith V., Yesodharan R., Noronha J.A., Ladd E., George A. (2021). Qualitative methods in health care research. Int. J. Prev. Med..

[25] Agius S.J. (2013). Qualitative research: Its value and applicability. Psychiatr. Bull..

[26] Allen A.P., Kennedy P.J., Cryan J.F., Dinan T.G., Clarke G. (2014). Biological and psychological markers of stress in humans: Focus on the Trier Social Stress Test. Neurosci. Biobehav. Rev..

[27] Allen A.P., Kennedy P.J., Dockray S., Cryan J.F., Dinan T.G., Clarke G. (2017). The Trier Social Stress Test: Principles and practice. Neurobiol. Stress.

[28] Kirschbaum C., Pirke K.M., Hellhammer D.H. (1993). The ‘Trier Social Stress Test’-a tool for investigating psychobiological stress responses in a laboratory setting. Neuropsychobiology.

[29] Birkett M.A. (2011). The Trier Social Stress Test protocol for inducing psychological stress. J. Vis. Exp..

[30] Salimetric’s SalivaLab (2018). Laboratory Report: Methodology.

[31] Touchette D.R., Stubbings J., Schumock G. (2012). Improving Medication Safety in High Risk Medicare Beneficiaries Toolkit.

[32] Cohen S., Kamarck T., Mermelstein R. (1983). A global measure of perceived stress. J. Health Soc. Behav..

[33] Haverman L., van Oers H.A., Limperg P.F., Houtzager B.A., Huisman J., Darlington A.-S., Maurice-Stam H., Grootenhuis M.A. (2013). Development and validation of the distress thermometer for parents of a chronically ill child. J. Pediatr..

[34] Marsh L., Warren P.L., Savage E. (2018). “Something was wrong”: A narrative inquiry of becoming a father of a child with an intellectual disability in Ireland. Br. J. Learn Disabil..

[35] Carter N., Bryant-Lukosius D., DiCenso A., Blythe J., Neville A.J. (2014). The use of triangulation in qualitative research. Oncol. Nurs. Forum..

[36] (2014). The Oxford Handbook of Qualitative Research.

[37] Lakshmanan A., Agni M., Lieu T., Fleegler E., Kipke M., Friedlich P.S., McCormick M.C., Belfort M.B. (2017). The impact of preterm birth < 37 weeks on parents and families: A cross-sectional study in the 2 years after discharge from the neonatal intensive care unit. Health Qual. Life Out..

[38] Grunberg V.A., Geller P.A., Patterson C.A. (2020). Utilization of NICU Infant Medical Indices to Classify Parental Risk for Stress and Family Burden. J. Pediatr. Health Car..

[39] Grunberg V.A., Geller P.A., Bonacquisti A., Patterson C.A. (2019). NICU infant health severity and family outcomes: A systematic review of assessments and findings in psychosocial research. J. Perinatol..

[40] Kersting A., Dorsch M., Wesselmann U., Lüdorff K., Witthaut J., Ohrmann P., Hörnig-Franz I., Klockenbusch W., Harms E., Arolt V. (2004). Maternal posttraumatic stress response after the birth of a very low-birth-weight infant. J. Psychosom. Res..

[41] Lasiuk G.C., Comeau T., Newburn-Cook C. (2013). Unexpected: An interpretive description of parental traumas’ associated with preterm birth. BMC Pregnancy Childbirth.

[42] Porges S.W. (2007). The polyvagal perspective. Biol. Psychol..

[43] Mazgelytė E., Chomentauskas G., Dereškevičiūtė E., Rekienė V., Jakaitienė A., Petrėnas T., Songailienė J., Utkus A., Aušrelė K., Karčiauskaitė D. (2021). Association of Salivary Steroid Hormones and Their Ratios with Time-Domain Heart Rate Variability Indices in Healthy Individuals. J. Med. Biochem..

[44] Hyde J., Ryan K.M., Waters A.M. (2019). Psychophysiological Markers of Fear and Anxiety. Curr. Psychiat. Rep..

[45] Smeets T. (2010). Autonomic and hypothalamic-pituitary-adrenal stress resilience: Impact of cardiac vagal tone. Biol. Psychol..

[46] La Marca R., Waldvogel P., Thörn H., Tripod M., Wirtz P.H., Pruessner J.C., Ehlert U. (2011). Association between Cold Face Test-induced vagal inhibition and cortisol response to acute stress. Psychophysiology.

[47] Maki P.M., Mordecai K.L., Rubin L.H., Sundermann E., Savarese A., Eatough E., Drogos L. (2015). Menstrual cycle effects on cortisol responsivity and emotional retrieval following a psychosocial stressor. Horm. Behav..

[48] Thayer J.F., Ahs F., Fredrikson M., Sollers J.J., Wager T.D. (2012). A meta-analysis of heart rate variability and neuroimaging studies: Implications for heart rate variability as a marker of stress and health. Neurosci. Biobehav. R.

[49] Griffith T., Singh A., Naber M., Hummel P., Bartholomew C., Amin S., White-Traut R., Garfield L. (2022). Scoping review of interventions to support families with preterm infants post-NICU discharge. J. Pediatr. Nurs..

[50] Melnyk B.M., Feinstein N.F., Alpert-Gillis L., Fairbanks E., Crean H.F., Sinkin R.A., Stone P.W., Small L., Tu X., Gross S.J. (2006). Reducing premature infants’ length of stay and improving parents’ mental health outcomes with the Creating Opportunities for Parent Empowerment (COPE) neonatal intensive care unit program: A randomized, controlled trial. Pediatrics.

